# Awareness and knowledge among dental teaching faculty of graphene in dentistry: Application

**DOI:** 10.6026/973206300211589

**Published:** 2025-06-30

**Authors:** Swapna Basimi, Swathi Devi Enaganti, Lakshmana Rao Bathala, Narayana Chava, Vaishnavi Devi Majeti, Venkata Vaibhav Puvvada

**Affiliations:** 1 Department of Prosthodontics, Govt. Dental College and Hospital, Afzangunj, Hyderabad, Telangana, India; 2 Department of Prosthodontics, KLR's Lenora Institute of Dental Sciences, Rajanagaram, Rajamahendravaram, Andhra Pradesh, India; 3 Department of Prosthodontics, SB Patil Institute of Dental Sciences and Research, Bidar, Karnataka, India; 4 Department of Oral and Maxillofacial Surgery, KLR's Lenora Institute of Dental Sciences, Rajanagaram, Rajamahendravaram, Andhra Pradesh 533294, India

**Keywords:** Graphene, dental education, nanomaterials, awareness, dental faculty, curriculum integration

## Abstract

Graphene is a novel nanomaterial which garnered interest for its promising applications in dentistry due to its superior mechanical and antimicrobial properties. Therefore, it is of interest to the knowledge and awareness of graphene among dental faculty across India through a cross-sectional survey using a 14-item questionnaire. Among 407 respondents, most were Associate Professors, with a notable awareness of graphene's potential despite no current clinical use in their institutions. The findings highlight a knowledge base that supports future integration of graphene into dental curricula and practice. Expanding educational initiatives may facilitate its eventual clinical adoption.

## Background:

Over the past decade, nanomaterials have played an important role in enhancing the results of numerous dental treatments. Among them, graphene has proven to be a highly promising material because of its remarkable physical properties, such as high flexural strength, excellent appearance, resistance to surface wear abrasion and excellent biocompatibility, antimicrobial action and wear resistance when compared with most traditional dental materials [1, 2- 3]. Graphene, a two-dimensional (2D) nanomaterial based on carbon obtained from graphite, was found in 2004 by Novoselov and Geim, who were jointly awarded the Nobel Prize in 2010 for their pioneering research [4, 5]. Graphene is present in a number of forms, such as single-layer graphene, few-layer graphene, graphene oxide (GO), reduced graphene oxide (rGO) and graphene-computer-aided manufacturing (G-CAM)-a biopolymer utilized in CAD/CAM millable discs [6]. Graphene is the strongest and thinnest material ever discovered, with a distinct hexagonal lattice structure, frequently found in single-layer sheets of carbon [7]. It has varied applications within the field of dentistry as an antifungal and antibacterial agent, drug delivery device, bioactive scaffold for tissue engineering, oral biosensor, coating for dental implants, wound healing desensitizing agent and aid for nerve regeneration after maxillofacial surgery [8]. Although these promising attributes and uses, graphene has not been universally incorporated in clinical dental practice. Therefore, it is of interest to evaluate the awareness and knowledge of graphene and its application in dentistry among teaching faculty in different dental institutions in India.

## Methodology:

## Study design and population:

This was a descriptive cross-sectional survey study carried out among dental teaching faculty members from various Indian dental schools. Faculty members at various levels - Professors & HODs, Professors, Associate Professors and Assistant Professors.

## Questionnaire development and data collection:

A self-administered, structured, 14-question multiple-choice questionnaire was formulated using literature review and expert validation. The questionnaire consisted of three parts:

[1] Demographic Information (Gender, Designation)

[2] Graphene Awareness among Dentists

[3] Graphene Application Knowledge in Dentistry

The questionnaire was sent through Google Forms on different dental teaching networks and social media sites. The responses were gathered from 1 July 2023 to 31 August 2023.

## Ethical aspects:

The study got ethical approval from the Institutional Ethical Committee of Lenora Institute of Dental Sciences (File No: 04/IEC/LIDS/Faculty/2023, Dated: 17/06/2023). Informed consent was taken before participants answered the questionnaire.

## Statistical analysis:

The data gathered were imported into Microsoft Excel and analyzed with SPSS version 26. Descriptive statistics, chi-square tests and p-values were applied to assess differences in awareness and knowledge by faculty designations. A p-value of <0.05 was deemed statistically significant.

## Results:

This research evaluated the awareness and knowledge of graphene in dental teaching staff from various institutions, reviewing responses from 407 individuals. Results are organized under major categories, such as demographic features, awareness levels, knowledge of graphene applications and use in dental research and practice.

Table 1 (see PDF) indicates that 407 faculties responded to the survey, with a greater percentage of men (57.5%) than women (42.5%) and the faculty designation distribution was Professors & HODs (43), Professors (83), Associate Professors (149) and Assistant Professors (132). As shown in Table 2 (see PDF), knowledge of graphene differed widely across designations with the highest rate among Professors & HODs (95.3%), whereas prior knowledge was only reported by 43.4% of Professors and 56.4% of Associate Professors; however, interestingly, all the Assistant Professors were familiar, with statistical significance (p=0.022). Table 3 (see PDF) reveals that there is low awareness of graphene's dental use-fewer than 11.3% knew about its application in pulp and bone regeneration and 25.8% appreciated its antibacterial and anti-inflammatory properties, while there was much greater awareness of its application as an implant coating material (68.5%, p=0.008). Table 4 (see PDF) is noted for familiarity among specialties and the greatest familiarity is exhibited in implantology (40.5%) and prosthodontics (35.4%), contrasting with less in endodontics (22.7%) and periodontics (18.9%), proposing greater familiarity within prosthetic and implant-centric disciplines. But, as evidenced in Table 5 (see PDF), no institutions indicated active use of graphene in research or clinical practice, highlighting the essential awareness-application gap and prioritizing the need for targeted faculty development and research integration.

## Discussion:

Graphene is a conductive nanomaterial with high physical properties, such as a surface area of 2630 m²/g and compressive strength of 1100 GPa and it is far stronger than steel but lighter than aluminium [9]. Although graphene possesses such extraordinary qualities, merely 25% of the faculties involved in the current study knew about these properties [10]. There was also limited awareness regarding the use of graphene oxide in biomedicine, with only 27.5% aware of its application and only 11.3% aware of its use as a scaffold [11]. Several studies have shown graphene to possess powerful antibacterial and antibiofilm activity, especially when blended with silver or zinc nanoparticles and its incorporation into dental materials such as glass ionomer cements and implant coatings improves their antimicrobial activity [12]. Knowledge of graphene-coated dental implants, however, was reserved largely for senior faculty, even though literature reported their advantages in osseointegration, bone regeneration and antifungal resistance [13]. Research further corroborates the use of graphene in enhancing polymer hydrophilicity and promoting cell adhesion and growth [14]. Graphene-based products have indicated potential in drug delivery, regenerative dentistry and biosensor design, including the early detection of diseases by means of saliva analysis [15]. Additionally, G-CAM, a graphene-polymer composite product applied in prosthodontics, is underappreciated, with only 11.3% of faculty recognizing its clinical potential. Graphene oxide also exhibits potential to act as a sustained-release antibiotic carrier for serious dental infections and inhibit dentine demineralization [15]. These results highlight the discrepancy between scientific progress and teacher-level awareness and it is stressed that more integration of graphene-related information into dental practice and education is required.

## Conclusion:

Despite significant awareness about graphene among dental faculty, knowledge of its specific applications remains limited. The findings emphasize the need for structured faculty training, research collaboration and curriculum updates to integrate graphene-based biomaterials into dental education and practice. Future studies should focus on evaluating the impact of educational interventions on graphene-related knowledge and its clinical applications in dentistry.
